# Taming the flames: the role of emotional intelligence in controlling moral outrage among medical students

**DOI:** 10.3389/fmed.2025.1702128

**Published:** 2026-01-08

**Authors:** Shereen El Tarhouny, Hossam Abou Saif, Zeinab El Sawaf, Tayseer Mansour

**Affiliations:** 1 Health Professions Education Center, Ibn Sina National College for Medical Studies, Jeddah, Saudi Arabia; 2 Department of Medical Biochemistry, Faculty of Medicine, Zagazig University, Zagazig, Egypt; 3 Clinical Psychology, Ibn Sina National College for Medical Studies, Jeddah, Saudi Arabia; 4 Department of Pathology, Faculty of Medicine, Taibah University, Al Madinah, Saudi Arabia; 5 Department of Medical Education, Faculty of Medicine, Suez Canal University, Ismailia, Egypt; 6 Department of Family and Community Medicine and Medical Education, Faculty of Medicine, Taibah University, Saudi Arabia

**Keywords:** emotional intelligence, emotional regulation, ethics, medical students, moral outrage, professionalism

## Abstract

**Background:**

Emotional intelligence (EI) is increasingly recognized as a critical competency for medical students, enabling them to effectively regulate emotions, navigate complex ethical dilemmas, and maintain professional integrity. However, the specific role of EI in moderating moral outrage, a strong emotional response to perceived ethical violations, remains underexplored. Given the potential for unchecked moral outrage to destabilize professional conduct and compromise patient care, understanding how EI influences, particularly among medical students in Egypt.

**Methods:**

A cross-sectional study assessed Emotional intelligence and moral outrage control among 478 medical students in Egypt, using validated, adapted instruments: a 15-item short form of the Schutte Self-Report Emotional Intelligence Test (SSEIT) and a newly developed 10-item Moral Outrage Scale (MOS) based on situational vignettes relevant to medical training. EI scores were categorized as low (15–29) or high (30–45), while moral outrage control was grouped as low (≤18) or high (19–30). Statistical analyses included frequencies, percentages, and chi-square tests to examine associations between EI and moral outrage regulation.

**Results:**

The majority of participants (79.92%) exhibited high emotional intelligence. A statistically significant positive correlation was found between emotional intelligence and moral outrage control (*r* = 0.62, *p* < 0.001). Regression analysis shows EI predicted 39% of the variance in moral outrage control (R^2^ = 0.33, *p* < 0.001). Students with high EI demonstrated significantly better capacity to regulate responses to ethically challenging situations.

**Conclusion:**

This study found that Emotional intelligence plays a significant role in shaping how medical students manage moral outrage. Medical students with higher emotional intelligence (EI) exhibited a significantly greater ability to regulate moral outrage, demonstrated by more controlled and constructive responses to ethical challenges. Integrating EI training into medical curricula could enhance ethical sensitivity and reduce the risk of maladaptive emotional responses in clinical practice. This study utilized self-reported measures, employed a cross-sectional design that restricts the ability to draw causal conclusions, and involved participants from a limited number of institutions, which may impact the generalizability of the findings.

## Introduction

Emotional intelligence (EI) has gained increasing recognition as a critical competency in modern healthcare and medical education, influencing. Communication, empathy, ethical decision-making, and professional conduct (1, 2). It is broadly defined as the ability to perceive, recognize, understand, regulate, and use emotions effectively for self and others (3, 4). Moreover, EI also fosters resilience and coping skills essential for physicians as they build their professional identity (5). Among medical students, higher EI is associated stress regulation interpersonal adaptability, and clinical performance (6, 7), while also contributing to the regulation of moral emotions such as moral outrage (8).

Research consistently demonstrates that EI integration in healthcare settings yields measurable improvements in patient outcomes, provider well-being, and institutional effectiveness (9). Medical institutions increasingly incorporate structured EI training throughout undergraduate and graduate programs, utilizing evidence-based curricula that integrate longitudinal skill development. Many institutions adopt Goleman’s four-domain EI framework including self-awareness, self-management, social awareness, and relationship management, to provide comprehensive competency development (11). Healthcare organizations leverage EI training to enhance teamwork effectiveness, reduce interprofessional conflicts, and improve communication among healthcare teams. EI-focused interventions consistently result in more collaborative environments that prioritize patient safety and foster progressive dialogue among multidisciplinary teams. Research demonstrates that healthcare professionals with higher EI exhibit significantly greater resilience, more effective stress management capabilities, and reduced turnover rates. Comprehensive meta-analyses reveal significant associations between EI training and decreased burnout across various healthcare roles, with effect sizes ranging from moderate to large (10).

Emotional intelligence training enhances providers’ abilities to navigate cross-cultural interactions and deliver culturally sensitive care, contributing to health equity initiatives, and also improves anger management and reduces impulsive moral outrage, enabling clinicians to respond more thoughtfully to ethical challenges (12). Moral outrage is defined as the “anger provoked by the perception that a moral standard, usually a standard of fairness or justice, has been violated.” It is subjectively experienced as the feeling of anger and the motivation to restore morality and justice (13).

Key brain regions implicated include: amygdala, which is Central for emotional salience and reactivity, including anger and moral indignation. Prefrontal Cortex (PFC): especially the dorsomedial and orbitofrontal regions, which are involved in evaluating social and moral information, regulating emotional responses, and supporting moral reasoning. Moreover, Anterior Insula and Anterior Cingulate Cortex: involved in the experience and regulation of disgust, anger, and empathy, and in integrating emotional and cognitive information during moral judgment. Additionally, Brainstem and Periaqueductal Gray: associated with autonomic arousal and action readiness during anger states (14).

Medical students are frequently exposed to ethically challenging situations that may trigger moral outrage, an intense emotional reaction to perceived moral transgressions, ethical violations, or injustices (15). This is a frequent and complex experience in clinical training settings. Moral outrage, while a natural response to ethical breaches, can have both positive and negative consequences. On one hand, it can motivate individuals to advocate for justice, ethical practice, and even catalyze whistleblowing (16, 17). On the other hand, uncontrolled outrage can lead to unproductive emotional reactions, stress, impaired decision-making, and reactive, counterproductive behaviors, especially when not properly managed (15, 18).

Medical students, who are still in the formative stages of their professional development, are particularly vulnerable to the destabilizing effects of unchecked moral outrage. They must learn to balance their emotional responses while maintaining ethical clarity and composure, especially when confronted with ethically challenging or unjust situations (19).

The capacity to regulate moral outrage constructively is closely linked to emotional competencies such as empathy, self-awareness, and emotional regulation, core components of EI (20–22).

Emotional intelligence (EI) is fundamental to established professional competency frameworks, underscoring its role in effective communication, collaboration, and ethical practice. Globally, the importance of EI is included in medical education standards. The Accreditation Council for Graduate Medical Education (23), for example, identifies Professionalism, Interpersonal and Communication Skills, and Systems-Based Practice as core domains that necessitate emotional regulation and sound ethical judgment (24). Likewise, the CanMEDS framework integrates these dimensions into the essential roles of Communicator, Professional, and Collaborator for compassionate medical practice. Concurrently, the UK’s General Medical Council (GMC) Outcomes for Graduates explicitly mandates that graduates possess emotional resilience, a commitment to patient-centered care, and moral integrity.

Regionally, Egypt’s NARS-Medicine 2017 framework reflects this international alignment by transitioning to a competency-based model that foregrounds professionalism, effective communication, collaboration, and ethical medical practice (25).

Evidence from systematic reviews and meta-analyses indicates that structured EI interventions improve empathy, teamwork, stress regulation, and clinical communication, all essential for professional identity formation and ethical practice (26–28). Despite this, the specific role of EI in moderating moral outrage among medical students remains underexplored, a critical gap given its implications for ethical decision-making and professional development (29). Building on international evidence, the present study aims to examine how emotional intelligence contributes to regulating moral outrage in medical students and to assess whether EI can serve as a predictor of moral outrage regulation.

## Methodology

### Study design

This descriptive cross-sectional study examined the relationship between emotional intelligence and moral outrage control among medical students across multiple Egyptian medical colleges during the 2024–2025 academic year. The investigation employed a multi-institutional approach to enhance generalizability and reduce potential institutional bias.

### Participants and sampling

Using stratified random sampling, 478 medical students were recruited from pre-clinical (years 1–3) and clinical (years 4–6) phases of medical education. A sample size calculation was performed using Open Epi version 3.01, based on a 95% confidence level, an expected frequency of 50%, and a 5% margin of error. The population was conservatively estimated at 10,000 students across participating institutions, yielding a minimum required sample of 370 participants. A 30% buffer was added to account for potential non-response rates, resulting in the final recruitment target of 478 participants. Inclusion criteria required participants to be currently enrolled full-time medical students, aged 18–25 years, and willing to provide written informed consent. Participation in the study was entirely voluntary, and no incentives, monetary or otherwise, were offered. Students were excluded if they had prior formal training in psychology or counseling, documented psychiatric conditions requiring ongoing treatment, or participation in concurrent psychological research studies. Data was collected electronically for over 3 months.

### Data collection instrument

#### Emotional intelligence assessment

A validated 15-item version of the Schutte (30) Self-Report Emotional Intelligence Test assessed three core domains: appraisal and expression of emotion, regulation of emotion, and utilization of emotion. Participants rated items on a 3-point Likert scale (1 = Disagree, 2 = Uncertain, 3 = Agree), yielding total scores ranging from 15 to 45 points. Scores were dichotomized using empirically derived cutoffs established through Delphi consensus involving seven experts: 15–30 points indicated lower emotional intelligence, while 31–45 points represented higher emotional intelligence. The instrument demonstrated strong internal consistency (Cronbach’s α = 0.834).

#### Development of the Moral Outrage Scale

The Moral Outrage Scale (MOS) was designed to measure the regulation of moral outrage in ethically provocative contexts. A set of 10 situational vignettes was developed specifically for medical student populations, presenting ethically provocative vignettes that represent common dilemmas encountered in medical training. The situational vignette was designed to reflect realistic, non-clinical yet ethically relevant scenarios within the early phases of medical education. These scenarios focus on interpersonal and academic contexts that simulate typical moral conflicts faced by students, such as teamwork, perceived unfair treatment, breaches of academic integrity, and witnessing disrespectful behavior in academic settings.

These situations were intentionally chosen because they can elicit moral emotions and ethical judgments similar in structure to those experienced in clinical contexts, allowing for the assessment of moral outrage in a controlled, developmentally appropriate environment. This approach aligns with previous educational research emphasizing that early exposure to ethically and emotionally challenging academic situations contributes to the gradual formation of professional identity and ethical reasoning skills (19). The development process began with a comprehensive review of literature, expert panel consultation, adaptation of vignette-based items relevant to the target population, and integration of established frameworks for moral emotions [e.g., (31, 32)].

#### Scoring of MOS

In designing the MOS response options, moral outrage regulation was considered as a range of behavioral responses to ethically challenging situations, ranging from avoidance or passivity to balanced, assertive engagement, and ultimately, uncontrolled or confrontational reactions. Assertive responses were therefore conceptualized as adaptive regulation of moral outrage, whereas highly confrontational options reflected dysregulated expression.

Each vignette included three response options representing qualitatively distinct behavioral categories rather than degrees of agreement. Different levels of moral outrage control include

- Confrontational responses (1 point): these indicate impulsive expression of moral outrage, as this represents poor control, and they were assigned the lowest score
- Avoidance responses (2 points), these represent emotional inhibition or withdrawal. Although avoidance prevents confrontation, it does not reflect optimal emotional regulation. Therefore, it was assigned a mid-level score.
- Controlled assertive responses (3 points). These reflect the highest level of moral outrage control. Such responses involve recognizing ethical tension, maintaining emotional calm, and choosing a professional, constructive course of action. As these align with effective regulation of moral emotion, they were assigned the highest score.

Cut-off values for categorizing overall levels of moral outrage control were allocated based on the theoretical expectation that controlled responses represent the optimal behavioral benchmark in ethics-related emotional management.

This scoring structure ensures that higher scores consistently represent stronger moral outrage regulation. Total scores ranged from 10 to 30 points, with scores of 10–20 classified as lower moral outrage control and 21–30 as higher control.

#### Validation of MOS

To ensure the content validity of the 10-item Moral Outrage Scale (MOS), a formal validation was conducted with a panel of six experts in medical ethics, Professionalism, and psychology.

They independently rated each scenario for relevance, clarity, realism, and ability to elicit moral outrage using a 4-point relevance scale (1 = not relevant, 2 = somewhat relevant, 3 = quite relevant, 4 = highly relevant).

Item-Level Content Validity Index (I-CVI) was computed for each item, representing the proportion of experts who gave a rating of 3 or 4. Values ranged from 0.80 to 0.93. All items met or exceeded the established criterion of 0.78 for a panel of this size, confirming their relevance.

Additionally, the overall Scale-Content Validity Index (S-CVI/Ave), was 0.91, surpassing the 0.90 threshold for excellent content validity based on Shi et al. (33).

Item clarity, contextual relevance, and cultural appropriateness were refined based on expert feedback, ensuring vignettes realistically reflect challenges medical students face.

For internal reliability, pilot testing with 20 students demonstrated acceptable reliability (Cronbach’s α = 0.711), indicative of acceptable psychometric performance.

#### Ethical considerations

All procedures received IRB approval from the lead institution (Suez Canal University) (Protocol 6123#) with the understanding that the standardized, anonymous protocol would be implemented across all collaborating institutions.

Written informed consent, and anonymous data collection protocols. Confidentiality was maintained throughout the research process.

### Statistical analysis

Data were analyzed using SPSS version 25.0. Descriptive statistics (frequency, mean, standard deviation) were used to summarize demographic variables and overall, EI and moral outrage scores. Pearson’s correlation coefficient (r) was used to assess the relationship between emotional intelligence and moral outrage control. A linear regression analysis was conducted to determine whether emotional intelligence significantly predicted the ability to regulate moral outrage. Independent sample *t*-tests were used to assess gender and age-related differences in both variables. A *p*-value of <0.05 was considered statistically significant.

## Results

### Participant demographic characteristics

A total of 478 medical students participated, representing a robust sample that exceeded the minimum requirements. Participants were recruited through convenience sampling, with the survey link distributed across various online platforms. Consequently, it was not possible to determine a precise denominator or calculate a formal response rate. The predominant age group comprised students aged 20–25 years (50%), with those under 20 years constituting a similar proportion (46.86%). Females represented the majority of the sample (60.88%), and first-year students were the most represented academic cohort (51.88%), as summarized in Table 1.

**TABLE 1:** Sociodemographic characteristics of participants (*n* = 478).

Variable	Category	Frequency	Percentage (%)
Age (years)	<20	224	46.86
	20–25	239	50.00
	26–30	15	3.14
Gender	Male	187	39.12
	Female	291	60.88
Year of study	1st	248	51.88
	2nd	40	8.37
	3rd	94	19.67
	4th	79	16.53
	5th	12	2.51
	6th	5	1.05

**FIGURE 1:**
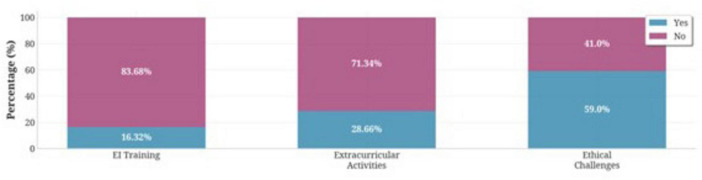
Participation rates in emotional intelligence training, extracurricular activities, and exposure to ethical challenges among medical students (*N* = 478).

Professional development backgrounds revealed that 16.32% had received formal emotional intelligence training, while 28.66% engaged in related extracurricular activities. Notably, 59.0% reported prior encounters with ethically challenging situations during medical training (Figure 1).

### Emotional intelligence distribution

Assessment results in Table 2 revealed predominantly high self-reported emotional intelligence levels, with 382 students (79.92%) classified as having high emotional intelligence among study participants and 96 students (20.08%) categorized as having lower emotional intelligence.

**TABLE 2:** Frequency and percentage of responses to the emotional intelligence scale.

No	Question	Response category	Frequency	Percentage
1	I know when to speak about my personal problems to others	Agree	367	76.78
		I don’t know	61	12.76
		Disagree	50	10.46
2	Other people find it easy to confide in me	Agree	323	67.57
		I don’t know	75	15.69
		Disagree	80	16.74
3	I am aware of my emotions as I experience them	Agree	323	67.57
		I don’t know	83	17.36
		Disagree	72	15.06
4	I am aware of the non-verbal messages I send to others	Agree	250	52.30
		I don’t know	82	17.15
		Disagree	146	30.54
5	By looking at their facial expressions, I recognize the emotions people are experiencing	Agree	178	37.24
		I don’t know	110	23.01
		Disagree	190	39.75
6	I easily recognize my emotions as I experience them	Agree	364	76.15
		I don’t know	59	12.34
		Disagree	55	11.51
7	I present myself in a way that makes a good impression on others	Agree	371	77.62
		I don’t know	50	10.46
		Disagree	57	11.92
8	When I am faced with obstacles, I remember times I faced similar obstacles and overcame them	Agree	141	29.50
		I don’t know	95	19.87
		Disagree	242	50.63
9	When my mood changes, I see new possibilities	Agree	321	67.15
		I don’t know	62	12.97
		Disagree	95	19.87
10	I have control over my emotions	Agree	334	69.87
		I don’t know	91	19.04
		Disagree	53	11.09
11	I help other people feel better when they are down	Agree	329	68.83
		I don’t know	80	16.74
		Disagree	69	14.44
12	I seek out activities that make me happy	Agree	338	70.71
		I don’t know	61	12.76
		Disagree	79	16.53
13	When I am in a positive mood, I am able to come up with new ideas	Agree	186	38.91
		I don’t know	64	13.39
		Disagree	228	47.70
14	I motivate myself by imagining a good outcome to tasks I take on	Agree	296	61.92
		I don’t know	59	12.34
		Disagree	123	25.73
15	I compliment others when they have done something well	Agree	297	62.13
		I don’t know	86	17.99
		Disagree	95	19.87

Item-level analysis revealed specific strengths and development areas. Highest agreement rates occurred for interpersonal and self-presentation items, for instance, 77.62% agreed they “present themselves well to make good impressions,” 76.78% “know when to speak about personal problems,” and 76.15% “easily recognize emotions as experienced.” Students demonstrated confidence in seeking positive experiences (70.71%) and emotional self-regulation (69.87%).

However, certain aspects showed limited endorsement, revealing potential development opportunities. Only 29.50% agreed they “remember overcoming similar obstacles when faced with challenges.” Additionally, just 38.91% agreed they “can come up with new ideas when in positive moods.”

### Moral outrage control assessment results

Table 3 shows moral outrage control assessment that 311 participants (65.1%) demonstrated high levels of control, while 167 students (34.9%) exhibited lower control levels. Scenario-specific analysis revealed considerable variability in responses across different ethical contexts. In academic integrity scenarios, such as observing cheating during exams, 56.28% indicated they would report or directly confront the behavior, while only 12.55% would ignore it, demonstrating strong moral engagement. Professional conduct scenarios elicited more varied responses, with 49.58% reporting they would become visibly upset and demand apologies for inappropriate comments during clinical sessions.

**TABLE 3:** Frequency and percentage of responses to controlling moral outrage scenarios.

Scenario and response options	Frequency	Percentage (%)
**1. During a discussion, your colleagues are stubbornly insisting on their opinions and dismissing yours.**		
A. I listen quietly without participating further.	57	11.92
B. I engage in discussion and try to present my perspective respectfully.	121	25.31
C. I argue my position forcefully and refuse to yield until they agree with me.	300	62.76
**2. I observe a colleague treating other colleagues unfairly.**		
A. I ignore the situation and do not get involved.	40	8.37
B. I try to mediate and restore harmony between them.	301	62.97
C. I confront the colleague who was unfair and insist they apologize	137	28.66
**3. While studying with friends, one friend is distracting the group and wasting time.**		
A. I go along with the distraction.	195	40.79
B. I try to encourage my friend to focus on studying.	135	28.24
C. I firmly stop my friend and insist we return to studying.	148	30.96
**4. A colleague insults or gossips about a friend who is not present.**		
A. I remain silent and do not react.	134	28.03
B. I calmly express that this behavior is inappropriate.	183	38.28
C. I strongly reject this behavior and leave the group angrily.	161	33.68
**5. A friend tries to show you movies or videos you find inappropriate or offensive.**		
A. I politely decline and avoid confrontation.	153	32.01
B. I explain my discomfort and ask not to be shown such content.	233	48.74
C. I strongly object and warn my friend not to repeat this behavior.	92	19.25
**6. During a mixed-gender lecture, a colleague behaves in a way that seeks inappropriate attention.**		
A. I laughed off the behavior.	212	44.35
B. I privately express my disapproval to the colleague.	151	31.59
C. I openly confront the colleague and demand they stop.	115	24.06
**7. You notice a colleague attempting to cheat during an exam.**		
A. I ignore the behavior.	60	12.55
B. I quietly ask my colleague not to cheat.	149	31.17
C. I report the incident or confront my colleague directly.	269	56.28
**8. During a practical/clinical session, some colleagues make unethical or inappropriate comments.**		
A. I ignore the comments	42	8.79
B. I discreetly address the behavior with the colleague	199	41.63
C. I become visibly upset and insist on an apology.	237	49.58
**9. A colleague repeatedly makes jokes during a lecture, disrupting the class.**		
A. I focus on the lecture and ignore the jokes	42	8.79
B. I ask the colleague to stop joking so we can concentrate.	165	34.52
C. I blamed the colleague sharply and warned them not to do it again.	271	56.69
**10. How do you generally see yourself in situations involving ethical challenges?**		
A. I tend to avoid conflict and stay uninvolved.	128	26.78
B. I respond in a balanced and typical manner, like most people.	244	51.05
C. I hold myself and others to high standards and intervene strongly when needed.	106	22.18

Interpersonal conflict scenarios revealed additional complexity (Scenario 1), with 62.76%. Social situations presented different challenges (Scenario 3), as only 30.96% would firmly redirect friends wasting study time, while 40.79% would accommodate distractions.

### Association between emotional intelligence and moral outrage control

Chi-square analysis revealed a statistically significant association between emotional intelligence and moral outrage control (χ^2^ = 7.54, *p* = 0.006), indicating meaningful relationships between these constructs.

**FIGURE 2:**
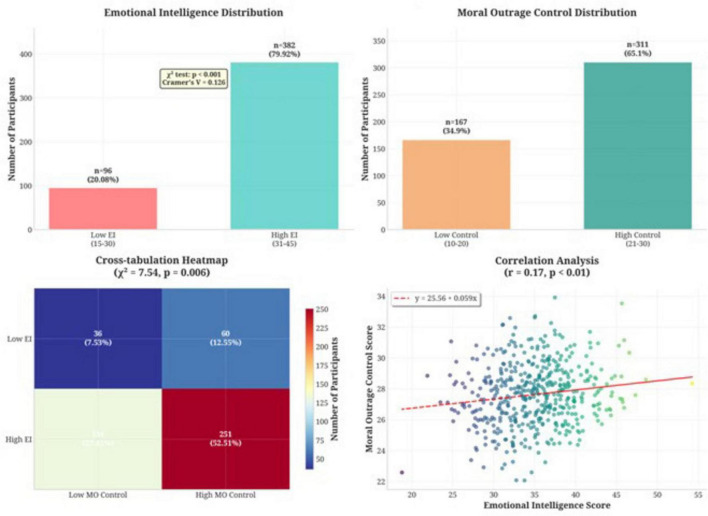
Relationship between emotional intelligence and moral outrage control among medical students (*N* = 478).

Cross-tabulation analysis (Figure 2) revealed specific patterns illuminating this relationship. Among students with low emotional intelligence, 36 individuals (7.53%) also demonstrated low moral outrage control, while 60 students (12.55%) exhibited high control despite low emotional intelligence. Conversely, among high emotional intelligence students, 131 individuals (27.41%) showed low moral outrage control, while 251 students (52.51%) demonstrated both high emotional intelligence and high moral outrage control.

### Predictive relationship and regression analysis

Linear regression analysis confirmed that emotional intelligence significantly predicts moral outrage control (*F* = 14.85, *p* < 0.01). The regression equation: Moral Outrage Control = 24.76 + 0.08 × (Emotional Intelligence Score) indicates that each one-point increase in emotional intelligence predicts a 0.08-point increase in moral outrage control.

The multiple correlation coefficient (R) between the variables was 0.17, indicating a positive but modest contribution of the independent variable to the prediction of the dependent variable. The coefficient of determination (R^2^) of 0.33 means that 33% of the variance in the dependent variable is explained by the model. Although this represents a small effect size, the relationship is statistically significant and practically meaningful, especially given the complexity of psychological constructs which are typically influenced by multiple factors. The standardized regression coefficient (β = 0.17) indicates that a one standard deviation increase in emotional intelligence is associated with a 0.17 standard deviation increase in moral outrage control (Table 4).

**TABLE 4:** Regression between emotional intelligence and controlling moral outrage among medical students.

Variables	R	R square	Constant	B	Beta	F	t
Emotional intelligence	0.17	0.33	24.76	0.08	0.17	14.85**	3.85**

**Significant at the level (0.01).

## Discussion

This study affirms that emotional intelligence (EI) significantly impacts medical students’ ability to control moral outrage in Egyptian medical colleges. The findings reinforce the idea that EI is central to ethical self-regulation and professional development in medical training and contribute to the growing body of literature examining psychological competencies essential for professional development in healthcare education.

### Emotional intelligence levels and patterns

The predominance of high emotional intelligence levels among medical student participants (79.92%) suggests that most students possess strong abilities in recognizing, understanding, and managing their own emotions as well as those of others, traits essential for effective communication and professional conduct, especially in medical schools’ demanding environments.

Our findings align with existing literature that medical students, by the nature of their academic and clinical environment, tend to develop higher emotional awareness and interpersonal competence (34, 35). A higher prevalence compared to some previously reported studies, yet it remains consistent with the upper spectrum of international data. For instance, Daud et al. (34) reported 57.5% of Malaysian medical students demonstrating high emotional intelligence, while Bashair et al. (36) found a rate of 69.1% in Palestinian cohorts. Additionally, Bitar et al. (37) reported a mean global trait EI score of 5.33 among Swedish medical students, suggesting comparable high-range values in certain European settings. This consistency across different educational and cultural contexts suggests that medical students generally possess strong foundational emotional awareness and regulation capabilities, which may reflect both self-selection into the medical profession and the influence of medical education experiences. Additionally, Medical schools attract individuals with inherently higher interpersonal competencies who are drawn to caring professions (38).

The item-level analysis for EI scale revealed specific strengths and development opportunities that warrant careful consideration. Students demonstrated confidence in interpersonal presentation skills (Q7) (77.62% agreement) and emotional self-awareness (76.15% agreement) (Q6), indicating strong foundational competencies for patient interaction and professional communication. These findings are consistent with research emphasizing the critical role of emotional intelligence in healthcare professional development, where effective patient-provider relationships depend heavily on emotional awareness and the appropriate expression of emotions (2).

However, the relatively lower endorsement of items related to resilience-building, such as overcoming obstacles (Q10) (29.50%), and creative problem-solving, such as generating ideas when in a positive mood (Q13) (38.91%), highlights critical gaps in students’ emotional skillsets that warrant targeted educational attention. These findings resonate with growing global concerns surrounding stress management and adaptability within medical training, particularly in light of the increasing prevalence of burnout and mental health issues among medical students (39).

The ability to leverage past experiences and positive emotions for resilient, adaptive problem-solving is a foundational competency in modern clinical settings (40). Structured emotional intelligence training could therefore bridge these skill gaps, enhancing student well-being and future professional performance.

### Moral outrage control and ethical decision-making

Roughly two-thirds (65.1%) of the participants exhibited high control over moral outrage, indicating a strong capacity to regulate emotional responses to ethical challenges or provocative situations.

Emotionally intelligent individuals may be better equipped to handle moral challenges by recognizing rising outrage before it escalates, a process neurologically associated with the insula and ventromedial prefrontal cortex (vmPFC). This early awareness allows for a cognitive shift from reactive, amygdala-driven responses to more reflective, prefrontal cortex-mediated reasoning. Such emotional regulation is crucial for maintaining professional conduct and ensuring fair, non-impulsive decision-making [(9), (41)].

This finding is particularly significant given that 59% of participants reported prior encounters with ethically challenging situations during their medical training, indicating that the majority had already faced real-world applications of these regulatory capabilities.

The scenario-specific analysis revealed important nuances in students’ ethical reasoning and emotional regulation across different contexts. The strong response to academic integrity violations (Scenario 7) (56.28% would report or confront cheating) demonstrates appropriate moral engagement and suggests that students recognize their professional obligations regarding academic honesty. This finding aligns with recent research on moral reasoning in healthcare education, which emphasizes the importance of developing strong ethical foundations during training (42, 43).

However, the more varied responses to professional conduct scenarios, particularly the tendency toward emotional reactivity (Scenario 8) (49.58% would become visibly upset at inappropriate comments), suggest that students may benefit from additional training in professional communication and conflict resolution. Recent literature emphasizes the importance of measured professional responses in healthcare settings, where emotional regulation directly impacts patient care quality and team dynamics (11). The finding that many students would argue forcefully when their opinions are dismissed (62.76%) (Scenario 1) further supports the need for enhanced training in professional communication and collaborative decision-making.

The social context findings, where students showed less assertiveness in redirecting peers during mixed gender study sessions (24.06) (Scenario 6), highlight the complex interplay between social relationships and moral courage. This pattern suggests that peer relationships may complicate students’ ability to maintain appropriate boundaries and assert their values, a finding that has important implications for team-based learning environments and future collaborative practice settings (44).

### Relationship between emotional intelligence and moral outrage control

The statistically significant association between emotional intelligence and moral outrage control (χ^2^ = 7.54, *p* = 0.006) underscores EI’s protective role in emotionally charged ethical situations and provides empirical support for theoretical models linking emotional competencies to ethical behavior.

The ability to control moral outrage depends on the dynamic interplay between limbic regions (generating emotional responses) and prefrontal regions (regulating and contextualizing those responses). High EI individuals are more effective at engaging these regulatory circuits, allowing them to experience outrage without losing cognitive control or acting impulsively. Conversely, dysfunction or underdevelopment in these networks (e.g., due to brain injury or poor emotional skills) can lead to excessive, poorly regulated moral emotions (45). Individuals with higher EI are better able to recruit prefrontal regions to modulate limbic (emotional) responses, resulting in more adaptive management of anger and moral outrage. This neural regulation helps prevent impulsive or excessive reactions to moral violations, supporting measured, prosocial behavior (46).

This relationship supports earlier findings by Guo et al. (28), who identified EI as a mediating factor in how healthcare professionals communicate and de-escalate emotionally intense interactions, also with other research by Mehralian et al. (47), who demonstrated that emotional and moral intelligence are vital in improving interpersonal relationships, particularly in medical and nursing fields, and that these competencies contribute to better decision-making and professional behavior. Furthermore, Lönn et al. (48) emphasize that emotionally challenging experiences, if adequately processed, can catalyze growth in professional identity and ethical sensitivity. Our study supports this by revealing that exposure to ethical dilemmas, while potentially distressing, also presents opportunities for self-awareness and value clarification, especially among those with higher emotional intelligence.

This finding is consistent with contemporary understanding of ethical decision-making as a complex, multifaceted process influenced by individual, contextual, and situational factors. The cross-tabulation analysis reveals that 27.41% of students with high emotional intelligence still demonstrated low moral outrage control, which reflects the distinct yet overlapping neural architectures underlying these two competencies, underscoring the complexity of this relationship and suggesting that emotional awareness alone is insufficient for optimal ethical behavior regulation. The finding that 52.51% demonstrated both high emotional intelligence and high moral outrage control suggests these competencies often co-occur and may be mutually reinforcing. However, 27.41% with high emotional intelligence but low moral outrage control indicates that emotional awareness and regulation skills do not automatically translate into effective moral outrage management (49).

Emotional intelligence and moral emotion processing involve interconnected neural systems. The prefrontal cortex regulates impulses, while limbic regions such as the amygdala shape emotional intensity (50). Social-cognition areas, including the temporoparietal junction, support moral judgment and perspective-taking. These mechanisms explain why individuals with higher emotional intelligence are better able to regulate morally charged emotions Yoder and Decety (51). Fortunately, EI-focused training can strengthen these regulatory pathways by enhancing self-awareness and impulse control. Such training may also improve ethical decision-making and emotional stability in clinical contexts (26).

Emotional intelligence development is a dynamic, trainable skill shaped through reflective practice, feedback, and social interaction. By understanding EI as an adaptive learning process that embeds structured opportunities for students to engage with ethical dilemmas, navigate peer collaboration, and reflect on their reactions to challenging situations, curricula better prepare learners for the complex demands of clinical practice. This approach fosters a professional identity that integrates emotional intelligence with sound moral judgment, ultimately supporting high standards of patient care and teamwork (26).

The regression analysis, which was statistically significant (*F* = 14.85, *p* < 0.01), indicates that emotional intelligence constitutes one element within a wider array of factors influencing ethical behavior. This finding aligns with recent theoretical developments in moral psychology, which emphasize the multifactorial nature of ethical decision-making and the importance of considering cognitive, emotional, social, and contextual influences (52).

The variability in scenario-based responses further reflects the “double-edged sword” nature of emotions in clinical education, as described by Toufan et al. (27). While emotions such as outrage may heighten moral vigilance, without adequate emotional processing, they can also result in impulsivity or professional disengagement. Our data revealed that while many students expressed assertive rejection of unethical behavior (e.g., peer cheating), others defaulted to passive or emotionally charged responses depending on the scenario.

The significant correlation found in this study supports the theory that emotional intelligence provides essential skills for managing moral emotions, including outrage. High-EI individuals are better equipped to recognize, understand, and regulate their emotional responses to ethical violations, which is crucial for maintaining professionalism and effective decision-making in clinical settings. Underscores EI’s protective role in emotionally charged ethical situations.

The moral outrage control findings suggest that while students generally possess adequate regulatory capabilities, there are opportunities for enhancement, particularly in professional communication and conflict resolution. The integration of emotional intelligence training with ethics education may provide synergistic benefits.

While EI is valuable, its psychometric foundations have been debated, particularly regarding construct overlap with personality traits and cultural bias in self-report tools. Most EI frameworks and instruments are built around neurotypical norms for emotional expression, regulation, and empathy, which can unintentionally marginalize or misinterpret the emotional styles of neurodiverse learners, such as those with autism spectrum traits or ADHD. This narrow framing risks pathologizing difference rather than recognizing that neurodivergent individuals may show distinctive emotional strengths, including deep empathy, heightened self-awareness, or strong moral sensitivity. That conventional EI models often fail to capture, as these models do not fully encompass the breadth of human emotional experience (53).

Therefore, both EI training and assessment must refrain from imposing narrow, normative models of emotional competence. Instead, curricula and support interventions should be tailored to recognize and nurture diverse emotional capacities and coping styles, ensuring accessibility and relevance for all learners.

While EI development offers value for most learners, it should not be seen as synonymous with moral virtue or as the sole criterion for ethical and professional competence. Rather, promoting inclusive, context-sensitive emotional education will best support the success and well-being of a diverse student body (54).

Emotional intelligence integration and training in the medical curriculum should start as early as possible. In the early stages of medical training, emotional intelligence (EI) is often exercised within academic and peer-related contexts rather than clinical ones. Students encounter emotionally charged situations such as teamwork challenges, breaches of academic integrity, perceived unfairness in grading or feedback, and interpersonal conflicts. These experiences serve as formative opportunities for developing self-awareness, empathy, and emotional regulation, key EI competencies that underpin moral reasoning and professional identity formation (5). Situating EI development within these realistic early-training contexts highlights the need for intentional educational strategies that integrate reflection, feedback, and mentorship to foster emotional growth before students transition into clinical environments (26).

Early academic and peer-based emotional challenges play a key role in shaping medical students’ emerging professionalism and emotional regulation skills. These non-clinical situations, such as teamwork difficulties, perceived academic unfairness, and interpersonal conflicts, provide formative opportunities for students to practice managing emotionally charged responses in a low-risk environment. Developing these foundational competencies early supports a smoother transition into clinical training, where ethical complexity and emotional demands intensify, requiring more advanced moral reasoning and emotional control.

### Practical implications

These findings suggest that emotional intelligence should be addressed explicitly and longitudinally within the medical curriculum, not only as a trait to be measured but as a set of situational skills that can be coached and supported. EI-focused teaching can be embedded in early team-based learning, ethics and professionalism modules, debriefings after simulated or real clinical encounters, and targeted support for students who struggle with emotional regulation or moral distress. Programs should also adopt an inclusive approach that recognizes neurodiversity and avoids imposing narrow norms of “ideal” emotional expression, instead helping all students develop flexible, context-sensitive strategies for managing moral outrage, sustaining empathy, and maintaining well-being in demanding clinical environments.

### Limitations

Several limitations should be acknowledged when interpreting these findings. The cross-sectional design precludes causal inferences about the relationship between emotional intelligence and moral outrage control.

While the study utilized self-report questionnaires, the lack of a social desirability assessment restricts the ability to assess or mitigate potential response bias.

Social Desirability Bias: individuals tend to present themselves in a favorable light, consciously or unconsciously. On an EI scale, this translates to respondents selecting answers that depict them as more emotionally skilled, empathetic, and regulated than they might actually be. This methodological decision was made to reduce the burden on participants; however, future research should incorporate a validated social desirability measure to more effectively address this type of bias.

Moreover, the proportion of students classified as having “high EI” and “high moral outrage control” was determined using empirically derived cut-off points. We acknowledge that these categorizations are sensitive to the choice of cut-off and may not represent the true prevalence of these traits in broader populations.

### Future directions and research

Future longitudinal mixed methods design studies could be considered to examine how these competencies develop throughout medical training and their long-term impact on professional behavior and patient care outcomes.

Longitudinal studies would allow tracking how emotional competencies evolve from preclinical to clinical years, providing insight into when and how educational interventions exert the greatest impact. Meanwhile, mixed-methods approaches, integrating quantitative assessment with qualitative reflections, interviews, or situational analyses, would offer a more comprehensive understanding of how students interpret and apply EI in real ethical or interpersonal contexts.

### Recommendations

The study recommends that medical schools integrate emotional intelligence (EI) training into their curricula, especially focusing on regulating moral emotions. Scenario-based learning and peer-led programs should be used to develop ethical reasoning and emotional skills, e.g., mindfulness training, emotion regulation exercises, and moral reasoning training. Faculty should model emotionally intelligent behavior, and regular EI assessments should be implemented to support students needing help with emotional regulation. These steps aim to prepare future doctors to handle ethical challenges in healthcare with both empathy and sound judgment.

## Conclusion

This study provides clear evidence that emotional intelligence is a significant determinant of medical students’ capacity to regulate moral outrage in ethically charged situations. The finding highlights emotional intelligence as a foundational component of ethical behavior, emotional regulation, and professional identity formation in medical education. These results strongly support embedding emotional intelligence and moral emotional regulation training into the medical curriculum, positioning these competencies as an essential teachable element of professional development rather than inherent traits.

## Data Availability

The original contributions presented in this study are included in this article/supplementary material, further inquiries can be directed to the corresponding author.
